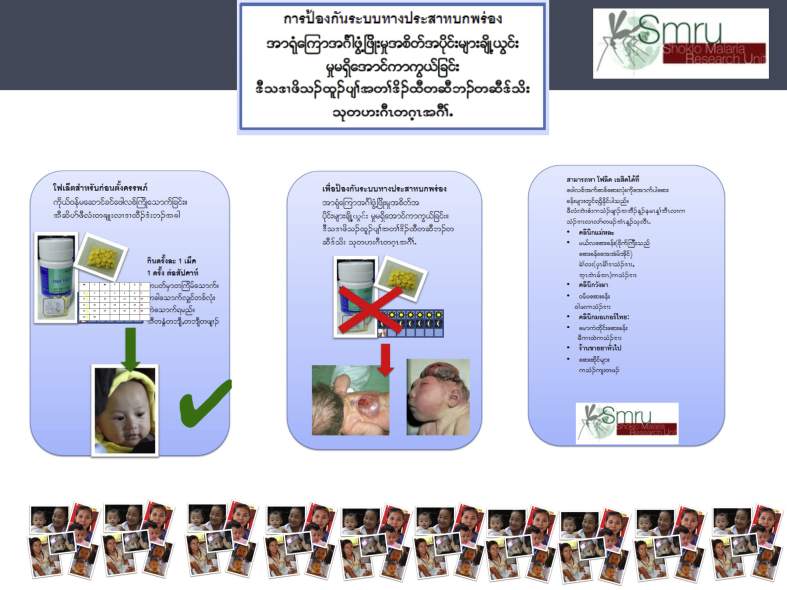# Folate supplementation to prevent birth abnormalities: evaluating a community-based participatory action plan for refugees and migrant workers on the Thailand-Myanmar border

**DOI:** 10.1016/j.puhe.2018.04.009

**Published:** 2018-08

**Authors:** A. Stevens, M.E. Gilder, P. Moo, A. Hashmi, S.E.T. Toe, B.B. Doh, S. Nosten, K. Chotivanich, Shawn Somerset, Rose McGready

**Affiliations:** a School of Allied Health, Faculty of Health Sciences, Australian Catholic University, Brisbane, Australia; b Shoklo Malaria Research Unit, Mahidol-Oxford Tropical Medicine Research Unit, Faculty of Tropical Medicine, Mahidol University, Mae Sot, Thailand; c Mahidol-Oxford Tropical Medicine Research Unit, Faculty of Tropical Medicine, Mahidol University, Bangkok, Thailand; d Centre for Tropical Medicine and Global Health, Nuffield Department of Clinical Medicine, University of Oxford, Oxford, United Kingdom

**Keywords:** Preconception folic acid, Neural tube defects, Thailand, Myanmar, Refugee, Migrant

## Abstract

**Objectives:**

Preconception folic acid (PFA) taken at least 3 months before conception can decrease the incidence of neural tube defects (NTDs) by approximately 46%. NTDs contribute significantly to neonatal morbidity and mortality in migrant and refugee populations on the Thailand-Myanmar border (incidence 1.57/1000 live births). This audit aimed to assess uptake of PFA among migrant and refugee women, evaluate knowledge about PFA among local healthcare workers and implement a participatory community intervention to increase PFA uptake and decrease NTD incidence in this population.

**Study design:**

A mixed-methods baseline evaluation was followed by an intervention involving health worker education and a community outreach program. A follow-up audit was performed 18 months post-intervention.

**Methods:**

Data were gathered via surveys, short interviews and focus group discussions. The intervention program included community-based workshops, production and distribution of printed flyers and posters, and outreach to various local organisations.

**Results:**

Uptake of PFA was <2% both before and after the intervention. Despite a substantial increase in local healthcare worker knowledge of PFA, no significant improvement in PFA uptake after the intervention was detected. Most pregnancies in this local community sample were reported to be unplanned.

**Conclusions:**

High rates of NTDs with low PFA uptake remains a major public health challenge in this transient population. Results indicate that improved health worker knowledge alone is not sufficient to enhance PFA uptake in this population. Integration of PFA education within expanded family planning programs and broad-based food fortification may be more effective.

## Highlights

• Audited preconception folic acid (PFA) campaign on Thai-Myanmar border.
• Low awareness of PFA among health workers and migrant/refugee pregnant women.
• Improved local health worker PFA knowledge after campaign and follow-up held after 18 months.
• Overall, campaign proved ineffective in increasing PFA uptake among pregnant women.

## Introduction

Preconception folic acid (PFA) taken at least 3 months before conception can prevent a significant proportion of congenital birth abnormalities caused by neural tube defects (NTDs)^1^ and cleft palate.^2^ PFA is safe and is recommended for all women of child-bearing age,^3^ either through fortification of food supply or supplements.

NTDs encompass a range of congenital abnormalities, from severe fatal defects to mild defects that nonetheless carry risk for severe disability.^4^ In low- and middle-income countries (LMICs), where surgical and rehabilitation services are often not available and educational and employment opportunities for those with disabilities are limited, even mild NTDs may have a profound impact on individuals, families and communities.^5^

High-income countries have seen an increase in PFA uptake as a result of awareness campaigns, with increases from baseline 2.4%–25.1% to post-campaign levels of 8.3%–53.5% in Norway, the Netherlands, the UK, Israel and Australia.^6^ Although awareness of PFA improved significantly, less than half of eligible women in most countries took PFA, and about half of those who did take it reported taking it incorrectly. Low rates of pregnancy planning have hampered the effectiveness of public awareness campaigns.^7^ To overcome these challenges, many countries have introduced population-based food fortification,^8^ which has been associated with a 46% decrease in NTDs.^4^

In LMICs, progress has been further impeded by poor health literacy and lack of resources.^4^, ^9^ One exception has been China, a country with anomalously high rates of pregnancy planning, where recommendations for PFA supplementation have shown marked population-level decreases in NTDs.^10^ However, even within the successful Chinese framework, being a migrant or moving between provinces was associated with poor PFA uptake.^11^

The need for PFA is particularly acute among refugee and migrant communities along the Thailand-Myanmar border. Health services for these communities are limited, as migrant and refugee populations lack access to either Thailand or Myanmar health systems. Health education campaigns targeting PFA are uncommon, and there is little food fortification other than targeted distribution of fortified rice flour in the refugee camps.^12^

Knowledge and uptake of PFA remains very low among Thai women of reproductive age,^13^ for whom deficiency has been demonstrated in both dietary intake and serum folate levels.^14^ Although refugee and migrant populations on the Thailand-Myanmar Border have NTD incidence of 1.57/1000 births, more than twice the rate in other areas in Thailand,^15^, ^16^ published reports of NTD prevalence or folic acid uptake in this region are lacking.

This study comprised a mixed-methods approach to develop and evaluate the impact of a community-based participatory action plan to enhance PFA uptake in these marginalised communities.

## Methods

### Setting

The Thailand-Myanmar border region is home to a culturally and economically diverse population including Thai, Karen, and Burmese. A large proportion of people who live and work along both sides of the border are migrants.

The Shoklo Malaria Research Unit (SMRU) has provided antenatal care and delivery services to the refugee population on the Thailand-Myanmar border since the mid-1980s and to the migrant population since 1998. At the start of the study, SMRU operated three clinics with a focus on maternal and child health: at Maela, WangPha and MawkerThai. Maela is the largest refugee camp in Thailand with an estimated population of 45,000 people who have fled an uncertain political and security situation in Myanmar. Antenatal care in Maela was transferred from SMRU to other humanitarian organisations (first the American Refugee Committee and then the International Rescue Committee) between the baseline and follow-up audits. WangPha and MawkerThai clinics serve migrant communities living along the Thailand-Myanmar border who face legal, financial and linguistic barriers to accessing health services in Thailand. Health facilities in Myanmar are prohibitive in cost and have variable standards of care, though positive changes are occurring.^17^ Maternal and child healthcare services provided at SMRU's clinics focus on free provision of early antenatal care, skilled attendance at birth, neonatal care and follow-up in infancy.^18^

### Study design, participant recruitment and data collection

The audit cycle included five components: setting standards for good quality practice, audit of current practice, feedback of findings and target setting, intervention to change practice, and repeat audit.^19^ The intervention involved the development and implementation of a community-based participatory action plan that was initiated in August 2015. The audit cycle, including cross-sectional surveys of pregnant women and local health workers, was conducted before the initial PFA campaign (July–August 2015) and repeated 18 months postintervention (February–March 2017). As SMRU was no longer active in Maela at the time of the follow-up audit, it was performed only at the migrant clinics.

Women seeking care at SMRU clinics who were currently pregnant or within 2 months postpartum and who willingly provided verbal consent were eligible for the study. Women are routinely given folic acid at antenatal care appointments, with a brief explanation, and most of the women surveyed had already received this routine care at the time of the survey. Patients were sampled sequentially with the aim to sample all women present at the antenatal clinic on a given survey day, although this was sometimes limited by the large numbers of patients or urgent clinical problems. The survey consisted of 13 questions related to PFA uptake (Additional file 1) and was repeated in a simplified form focused on PFA uptake 18 months after the intervention. Owing to low literacy levels in the patient population,^20^ surveys were conducted verbally by trained staff proficient in the participants' language(s) (Sgaw/Pwo Karen or Burmese). As part of the survey, staff showed women standard 5 mg folic acid tablets that have been used in this population for over 10 years, and prenatal vitamins commonly available for purchase locally and asked about recognition.

Local health workers (medics, midwives, nurses, ultrasound workers and basic healthcare workers) from each of the three sites were considered eligible if they were working at SMRU at the time of the audit. Purposive sampling of clinical staff reflected all departments and both new and experienced staff in the health worker interviews. The health worker survey comprised eight questions (Additional file 2) and was repeated 18 months after the intervention.

Focus group discussions (FGDs) among pregnant women explored knowledge, attitudes and practices relating to PFA in more depth. The same eligibility requirements applied for the FGDs as the surveys. Women were shown a flyer (Additional file 3) and researchers facilitated discussion about barriers to uptake and ideas for a public awareness campaign in the community. Written language on the flyer was limited and included Burmese, Karen and Thai.

Health workers and eligible women were informed that participation in any of these activities was voluntary and would not impact employment or future care at the clinic. Survey responses were transcribed and translated into English by trained translators and entered into SPSS for analysis. Additional anonymised demographic data on pregnant women were extracted from the clinic's electronic medical records.

### Public health intervention: community-based participatory action plan

The public health intervention, comprising a community-based participatory action plan funded by a $AUD 800 ($USD 568) donation, was implemented in August 2015. The campaign included workshops where the problem of NTDs and dosing of PFA were discussed with health workers, 20 posters (which were put on display in the main health centres and billboards in the refugee camp and at ethnic health organisation clinics outside the camp) and 10,000 colour pamphlets printed for distribution after explanation (e.g. at outpatient and antenatal clinics) to increase awareness and uptake of PFA. Seven community-based workshops were conducted over a 3-week period and included a number of stakeholders: SMRU and partner local health workers, women of childbearing age, men, non-governmental organisation and community-based organisation staff, refugee camp administrators, field managers and program coordinators. In each workshop, a research team member delivered a presentation covering NTD prevention through diet and supplementation and prevalence rates in the region, followed by small group discussions among participants to identify community responses to address the high rates of NTDs.

### Data analysis

Demographic data from baseline and follow-up populations were compared using Mann-Whitney U test for continuous variables or Chi-squared test for binary variables. Factors associated with PFA knowledge were evaluated by univariate analysis and changes in health worker knowledge were compared using Fisher's exact test. Data were analysed using SPSS for Windows, version 22.0, (SPSS, Inc., Chicago, Illinois, USA) and Stata, version 15, (StataCorp, College Station, Texas, USA). Qualitative data from FGDs were independently coded by themes by two researchers and summarised. Discrepant coding that could not be solved by discussion between the two researchers were presented to a third researcher for final coding.

## Results

### Audit

#### Women of child-bearing age

The baseline audit included 371 women from Maela, WangPha and MawkerThai, and the repeat audit included 307 women from WangPha and MawkerThai. Because of this, expected differences in demographics included refugee status, country of residence and ethnicity (Table 1). There were no other significant differences between the baseline and follow-up populations.

**Table 1:** Demographic characteristics of women participating in the baseline and follow-up surveys.

Characteristic	Baseline N = 371	Follow-up N = 307	*P*-value
Refugee, % (n)	37.7 (140)	0 (0)	<0.001
Age in years, mean [range]	25 [15–47]	25 [15–44]	0.48
Gravidity, median [range]	2 [1–10]	2 [1–9]	0.56
Primigravidae, % (n)	36.1 (134)	30.3 (93)	0.11
Primigravidae age in years, mean [range]	20 [15–37]	20 [15–35]	0.44
Parity (if parity ≥1), mean [range]	2 [1–8]	2 [1–7]	0.09
History abortion (if gravidity ≥1), % (n)	29.5 (70/237)	31.8 (68/214)	0.61
History stillborn (if parity ≥1), % (n)	1.4 (3/218)	2.5 (5/197)	0.39
Literate, % (n)	65.2 (242)	61.2 (188)	0.28
Ethnic group, % (n)			
Sgaw or Poe Karen	62.5 (232)	52.1 (160)	<0.001
Burman	27.8 (103)	40.7 (125)	
Burman Muslim	5.1 (19)	0.0 (0)	
Other (e.g. Mon, PaOh, Rakhine, Kachin)	4.6 (17)	7.2 (22)	
Country of residence, % (n)			
Thailand	70.4 (261)	58.0(178)	<0.001
Myanmar	29.7 (110)	42.0(129)	
Slept under a bednet last night, % (n)	97.0 (360)	Not asked	N/A

Before the intervention, 132/371 (35.6%) women surveyed were aware that folic acid tablets prevented NTDs, prevented brain abnormalities or improved the health of mother and baby. Despite this, only 5/371 (1.3%) women surveyed took folic acid before conception. An additional 77/371 (20.8%) knew folic acid was given for anaemia, and the rest either did not know what folic acid was or provided incorrect information, such as the belief that folic acid is for cold and flu, hypertension or to make childbirth easier.

There was no significant relationship between selected demographic variables (literacy, gravidity, country of residence or bednet use) and folic acid uptake or knowledge of the benefits of folic acid (Table 2). On the repeat audit, 2/307 (0.65%) patients reported taking folic acid before pregnancy (*P* = 0.465 compared with baseline). One of the two pregnant women who took PFA was a SMRU staff member.

**Table 2:** Odds ratios of factors associated with knowledge of folic acid in refugee and migrant women on the Thailand-Myanmar border at baseline survey.

Variable examined	N	PFA knowledge,^a^ % (n)	OR (95% CI)
Literate	242	36.4 (88)	Reference group
Illiterate	128	34.4 (44)	0.917 (0.585–1.436), *P* = 0.733
Myanmar side of border	110	28.2 (31)	Reference group
Thai side of border	260	38.8 (101)	1.619 (0.997–2.628), *P* = 0.058
Primigravida	133	34.6 (46)	Reference group
Not primigravida	237	36.3 (86)	1.077 (0.690–1.680), *P* = 0.821
Slept under a bednet last night	360	35.6 (128)	Reference group
Did not sleep under a bednet last night	10	40.0 (4)	1.208 (0.335–4.361), *P* = 0.749

PFA, preconception folic acid; OR, odds ratio; CI, confidence interval.

a Knowledge of folic acid is determined by the variable, what is folic acid used for, those who responded with healthy baby, healthy mother and baby, prevention of NTDs, NTDs+anaemia, prevent brain injury were grouped together, other participants were considered not to know that folic acid could be used to prevent NTDs or to conceive a healthy baby.

#### Local health workers

Health worker surveys before the intervention (*n* = 100) revealed that 85/100 (85%) had heard of NTDs but only 16/100 (16%) knew folic acid could be used to prevent NTDs when taken before conception. A further 24/100 (24%) knew it could be used but thought that it should be taken after a woman becomes pregnant. A smaller group, 10/100 (10%), thought that diet was the best way of preventing NTDs, and 47/100 (47%) could not identify a correct means of preventing NTDs.

Local health worker surveys 18 months after the intervention (*n* = 79) revealed 73/79 (93%) knew of NTDs (*P* = 0.162 compared to baseline) and 58/79 (73%) knew NTDs could be prevented by taking folic acid before conception (*P* < 0.001 compared to baseline). An additional six health workers knew that it should be taken before conception but did not know the reason why, making a total of 64/79 (81%) who knew when PFA should be initiated (*P* < 0.001 compared to baseline).

### Focus groups

Five focus groups with a total of 28 women of child-bearing age were conducted. Women were grouped according to shared language. The main themes that emerged were planning of pregnancy, the role of the clinic in folic acid distribution, word of mouth dissemination of information, the role of men and the role of organisations and community leaders.

#### Planning of pregnancy

Comments in focus groups about planning of pregnancies were consistent with the experience of local health workers: most of the participants reported that their current pregnancy was unplanned.

*It was a mistake. I didn't plan on another baby as I had had six already.* (*WangPha, Karen*)

In discussions around planning or accidental pregnancy, oral contraceptive pills (OCPs) were the only method of family planning mentioned. Some women volunteered that they had tried to prevent the current pregnancy with OCPs but the medicine was ineffective. These women, across multiple focus groups, attributed their pregnancy to expired medicine. This may represent a commonly held belief about OCP ineffectiveness, but there were no further explorations about women's understanding of other potential factors relating to OCP failure. Two women reporting planned pregnancies had successfully taken OCPs as prescribed until discontinuing when ready for conception.

*This is my third baby, but I wanted to have some time between pregnancies so my girl could have a younger brother, so I took pills for 2 years and then stopped taking it when I wanted to have this baby.* (*MawkerThai, Karen*)

*It was an accident. I already had two babies and didn't want another one, so I was taking pills, but maybe it was expired* (*MawkerThai, Burmese*)

One participant mentioned that family planning should be included in education about PFA.

*Tell people how to plan pregnancy and then also tell them how to take the [FA] pill.* (*Maela, Karen*)

#### The role of the clinic

When asked to brainstorm about strategies to disseminate information about PFA, women readily suggested clinic-based strategies. Women stressed the need for careful explanation by clinic staff to be sure that women understood PFA and took it correctly. This was often mentioned as a means of mitigating low literacy as a barrier to PFA uptake.

*Tell women who have a child at SMRU to tell others and get them to come to clinic if want baby in future.* (*MawkerThai, Karen*)

*I think that handing out flyers is ok, but women should see a nurse before getting the tablet.* (*Maela, Karen*)

However, some respondents also mentioned that clinic-based strategies may not be sufficiently far-reaching. Respondents said that the clinic may be too far for some women, and that they might prefer to access PFA from a pharmacy or village leader.

*Tell women where they can buy it. The clinic can sometimes be far, but if women know folic acid is important they will buy it from drug store.* (*WangPha, Karen*)

Women also noted an important limitation of clinic-based care that pregnant women rarely access reproductive health care before conception, underscoring the challenge that the lack of family planning poses to PFA uptake.

*I don't think people will come to clinic unless they are already pregnant.* (*Maela, Burmese*)

#### Word of mouth

Word of mouth was overwhelmingly the method most often suggested by participants to raise awareness and uptake of PFA in this community. Participants spontaneously volunteered themselves to spread the word in their own communities. Several mentioned that they were motivated to spread the word because they now understood the importance of PFA. They suggested that others, including pregnant women coming for antenatal care, would be similarly motivated once they grasp its importance.

*I will go back and tell my family about the importance. Everyone else will do the same, but only if it is explained to them.* (*MawkerThai, Burmese*)

One participant raised concerns that friends and family might not be reliable agents of community education.

*Good, but we need to explain to many women, as many cannot read. I am worried that it will not be explained properly by peoples' friends and family.* (*Maela, Karen*)

#### Role of men

Focus group facilitators asked specific questions about the potential role of men in an awareness campaign, which met with diverse responses. Most of the respondents proposed that involvement of men would have an impact on PFA uptake by close female family members. Some also mentioned that the men would talk to other men and village leaders, thereby multiplying the impact. A significant minority of women in each FGD were sceptical, expressing that men do not care about these issues or consider them to be ‘women's problems’.

*Talk to women in clinics and they will tell their friend, but also talk to husbands so that they can talk to village leaders, families and wives to tell people it is important.* (*WangPha, Karen*)

*Not sure, some men care, some don't care.* (*Maela, Burmese*)

*No, only women need to know.* (*MawkerThai, Karen*)

#### Organisations and community leaders

Involvement of schools, community-based organisations, churches and local authorities in potential PFA campaigns was suggested by participants, but secondary to clinic-based interventions. Section leaders (Maela) and village leaders were mentioned occasionally, but one participant expressed distrust of the camp leaders.

*Teach in school, talk to community organisations like KWO [Karen Womens Organisation] and they will help spread message.* (*WangPha, Karen*)

*Section leaders could also distribute, but some section leaders might also keep the drug and try to sell it.* (*Maela, Burmese*)

## Discussion

The community-based participatory action plan approach used in this intervention led to enhanced knowledge and awareness of the importance of PFA in NTD prevention among health workers. However, this enhancement did not translate into behaviour change in the form of enhanced PFA uptake in the target population. Difficulties in translating knowledge into practice have been described in other development contexts.^21^ The PFA uptake of about 1% in the present study is very similar to that reported in Thailand in 2007.^13^ The lack of improvement in PFA uptake in response to this intervention seems to be linked to the observation that most pregnancies in this population are unplanned, though causality cannot be proven. Even in high-income countries, unplanned pregnancy remains a significant barrier to PFA uptake, leading to the need for mandatory food fortification policies.^6^, ^7^, ^8^ As revealed in FGDs, clinic-based interventions for PFA uptake are not effective since most women only access the clinic after pregnancy is established, whereas folate supplementation is critical at or before conception. Local health worker education therefore has only limited potential to affect PFA uptake.

Low health literacy among women of child-bearing age in these communities was another barrier identified by women in the FGDs. Educational level has been shown to affect PFA uptake^6^ and influence success of health worker programs in LMICs.^22^ However, improved maternal and child health outcomes have been demonstrated among these populations despite persistent low literacy, showing promise for future campaigns for PFA.^20^ Further research is required to determine how these factors interact in this population.

While PFA uptake did not change over 18 months, health worker knowledge improved and key focus areas were identified, including health messaging in communities with low literacy and family planning. These findings have broader implications because initiatives by governments to promote preconception health and potentially improve women's health and pregnancy outcomes are needed. Prepregnancy care has the potential to improve pregnancy outcomes by impacting multiple health mediators including maternal nutrition (folic acid, iron, thiamine, overweight and underweight), behaviours (tobacco, betel nut and alcohol use), chronic conditions (obesity, hypertension and diabetes), infectious diseases (soil-transmitted helminths, malaria, tuberculosis and human immunodeficiency virus) and preventable diseases (vaccinations).^23^

Funding was only modest for this complex intervention, and it is unclear if this was a contributing factor to the absence of an impact. Despite the enthusiasm, concern and willingness of stakeholders to participate, intervention resources (posters, pamphlets and folic acid tablets) were limited, and impact may have been enhanced with extra resources. Timing, duration and repetition of the intervention may also have affected outcomes.

The data presented here suggest that a more comprehensive, sustained and resource-intensive strategy may be necessary to have a measureable impact on PFA uptake and consequent pregnancy outcomes. Effective solutions will likely need far-reaching community-level interventions that include folic acid fortification or supplementation strategies and appropriate and sustained messaging. There is a paucity of information on strategies for improving uptake of PFA through public health campaigns in LMICs. Whilst food fortification has worked in high-income countries, this relies on widespread consumption of processed food and is far less feasible in communities reliant on subsistence agriculture. However, an economic analysis from earlier this year showed that investing in folic acid fortification is cost-effective in developing countries.^9^ Given the similarly low rates of PFA across Thailand and Myanmar, collaborative efforts between the countries or within Association of Southeast Asian Nations (ASEAN) may raise the level of program success.

The issue of unplanned pregnancies emerged as the primary modifiable factor for this community. This study thus provides further evidence that effective community-level family planning initiatives remain key to nutritional status among women of child-bearing age with the potential to mediate adverse maternal and neonatal health outcomes.

## Author statements

### Acknowledgements

The authors would like to thank all of the women, healthcare workers, members of community-based organisations and ethnic health organisations and community members who participated in this work. They also thank the Federal Department of Education of Australia for its financial support.

### Ethical approval

As an audit to improve local practice, this work did not meet the criteria requiring ethical review. The audit was discussed with the local Tak Community Advisory Board, a committee of local representatives which assesses the relevance and appropriateness of research proposals for the local population. The Tak Community Advisory Board agreed that the work was useful and that verbal consent from participants was appropriate/sufficient (Ref 7/2/15).

### Funding

This work was supported by the Federal Department of Education (Short-Term Mobility Grant), Australia, and the Wellcome Trust of Great Britain (106698) (Major Overseas Programme—Thailand Unit Core Grant). The Shoklo Malaria Research Unit is part of the Wellcome Trust Mahidol University Oxford Tropical Medicine Research Programme.

### Competing interests

None declared.

## Figures and Tables

**figs1:**