# Supplementary material for: Folate supplementation to prevent birth abnormalities: evaluating a community-based participatory action plan for refugees and migrant workers on the Thailand-Myanmar border
Source: Public Health. 2018 Aug;161:83–9. doi: 10.1016/j.puhe.2018.04.009 (PMC6086336; doi:10.1016/j.puhe.2018.04.009)
Supplement: Multimedia component 1 [file mmc1.docx]

**Folate Knowledge Testing – Pregnant Women**

**Q1. Can you read**? (Show them the printed paper and ask them to read a sentence) **Y / N**

**Q2. How many weeks along where you when you first visited clinic? (***How many weeks pregnant, an estimate is ok, if Don’t know just write DK)*

**Q3. Did you sleep under a bed net last night? Y / N**

**Q4. Did you get a refugee ration in the first 3 months of pregnancy?** (Maela only) **Y / N**

**Q5.** **Did you receive the Asia Ration Mix in the first 3 months of pregnancy?** (Maela only) **Y / N**

**Q6. Do you know this tablet? (***Show pictures of folate to help explain what you mean, the answer should be Y or N, If yes go to next question.*) **Y / N**

**Q7 . Do you know what it is for?** *(Write down what they say, if they don't know, just write DK)*

**Q8. Did you take folate BEFORE YOU GOT PREGNANT THIS TIME?** *Y or N, if yes go to next question* ***Y / N***

***Q9.* When did you start taking it? (***Months before/ during pregnancy. E.g 10 weeks, one month before, three months before etc.)*

**Q10. How Many times a day or week did you take it?** (*One per day, twice per day, one per week etc.)*

**Q.11. where did you hear about it or who told you about it?(** *E.g MTC,BKK, MSH, MRMH, other?)*

**Q.12 Did you BUY IT or was GIVEN? Get it from there or somewhere else? Where?
Pay for It / Someone Gave to her Got it From Where** *(Clinic? Drug Store? SMRU? Etc_*

**Q.13 Have you told other women planning on becoming pregnant about folate? Y / N**
