# Supplementary material for: Folate supplementation to prevent birth abnormalities: evaluating a community-based participatory action plan for refugees and migrant workers on the Thailand-Myanmar border
Source: Public Health. 2018 Aug;161:83–9. doi: 10.1016/j.puhe.2018.04.009 (PMC6086336; doi:10.1016/j.puhe.2018.04.009)
Supplement: Multimedia component 2 [file mmc2.docx]

**Folate Knowledge Audit – SMRU Staff Date: ____/_____/_______**

**Q1. What do you do at SMRU?** *(Occupation)*

**Q2. Have you heard of folate or folic acid before(***Y or N, if yes go to next question)* **Y N**

**Q3. What is it for?**

**Q4. Folic Acid comes in tablets, but is also available in food. Name some foods that have folate?** *(if they don’t know just write DK)*

**Q5.** **At what stage of Pregnancy should a women take folate?** *(just write down what they say, if they don’t know DK)*

**Q6. Have you seen the SMRU Flyer about folate in pre-pregnancy?** (*Show them the Flyer*) **Y N**

**Q7. Have you heard of Neural Tube Defects? Y N**

***Q8.* What is one way of preventing Neural Tube Defects?** *(write down what they say)*
